# School-Based Enhanced Hearing Screening and Specialty Telehealth Follow-Up for Hearing Loss Among Children in Rural Alaska: Study Protocol for a Hybrid Effectiveness-Implementation Stepped Wedge, Cluster-Randomized Controlled Trial (North STAR Trial)

**DOI:** 10.21203/rs.3.rs-5252346/v1

**Published:** 2025-04-01

**Authors:** Samantha Kleindienst Robler, Janet Prvu Bettger, Elizabeth Turner, Alyssa Platt, David Arthur, Philip Hofstetter, Hannah Lane, Tarika Srinivasan, Shayu Deshpande, Matthew Hirschfeld, Susan D Emmett

**Affiliations:** University of Arkansas for Medical Sciences; Duke Global Health Institute; Duke Global Health Institute; Duke Global Health Institute; Duke Global Health Institute; Petersburg Medical Center; Duke University; Harvard Medical School; University of Arkansas for Medical Sciences; Southcentral Foundation; University of Arkansas for Medical Sciences

**Keywords:** Hearing loss, child, school health services, telehealth, health policy, rural health, implementation, healthcare access, stepped-wedge, cluster-randomized

## Abstract

**Background:**

Childhood hearing loss has well-known profound implications for language development, school achievement, and future employment opportunities. School-based health programs can provide hearing screening, but access to specialists for follow-up care is limited in rural areas. This is especially problematic for children in rural Alaska who experience a disproportionately high burden of preventable childhood hearing loss. The purpose of this study will be to develop and test the effectiveness and implementation of school-based specialty telehealth follow-up to improve timely access to specialty care after school hearing screening in rural Alaska.

**Methods:**

This will be a hybrid type 1 effectiveness-implementation stepped wedge, cluster-randomized trial in three representative regions of Alaska. The trial will evaluate the STAR model, which consists of three core components: 1) enhanced school hearing screening, 2) school-based specialty telehealth follow-up, and 3) streamlined communication between schools, healthcare providers, and parents/caregivers. The trial will begin with a formative phase in the first two years, when qualitative data on community preferences and perspectives will be gathered to systematically adapt the STAR model and develop an implementation plan for participating regions. The adapted STAR model will be evaluated with a stepped wedge, cluster-randomized design in approximately 25 schools in three regions of rural Alaska. The primary effectiveness outcome will be the proportion of referrals resulting in specialty follow-up within 60 days of school hearing screening, measured using queries of electronic health records from the healthcare systems serving each region. Generalized estimating equations (GEE) will be used to model these cluster-period school-level proportions to obtain population-averaged intervention effects that are of public health relevance and therefore of interest in implementation trials. Secondary implementation outcomes will include fidelity, reach, acceptability, feasibility, and appropriateness. Sustainability of the STAR model will be evaluated through iterative meetings with state leaders and policymakers.

**Discussion:**

This trial will evaluate school-based specialty telehealth follow-up in diverse regions of Alaska, addressing preventable childhood hearing loss with a model that could be translated to other underserved rural and marginalized groups to bring high-value services into rural schools and alter the paradigm of prevention nationwide.

**Trial registration:**

NCT05593484 - https://clinicaltrials.gov/study/NCT05593484

## Introduction

### Background and rationale {6a}

Childhood hearing loss leads to significant speech and language delays (1, 2). As a result, children with hearing loss perform worse in school, are more likely to drop out, and are 23–39% less likely to attend college (3–6). Hearing loss also has substantial psychosocial and behavioral implications, and children with even mild losses have lower quality of life and worse behavioral outcomes than peers with normal hearing (7–11). The World Health Organization estimates up to 60% of childhood hearing loss may be preventable, and this estimate rises to 75% in resource-constrained settings such as rural communities (12). Schools are an essential and unique access point to provide preventive services to rural children.

Prevalence of childhood hearing loss and middle ear disease is high in rural Alaska Native children, with our recent study indicating 10.5% prevalence of hearing loss and 17.6% prevalence of middle ear disease, compared to 1.7–5% prevalence in the general US (13, 14). The majority of hearing loss in Alaska is related to infection, arising from otitis media that is 4–5 times more prevalent in rural Alaska Native children than the general US despite pneumococcal vaccination (15–18). The infection-related hearing loss that is common in Alaska is considered preventable, in contrast to congenital etiologies. Although the state of Alaska mandates school hearing screening, current screening protocols in Alaska do not include a middle ear assessment (e.g. tympanometry), which can play an essential role in identifying preventable infection-related hearing loss.

A critical component after hearing screening is specialty follow-up, as the necessary monitoring and treatments provided by audiologists and otolaryngologists are not available in primary care. Follow-up care requires multiple steps. In our previous trial, 68% of children receiving standard referral (letter home to parents after school hearing screening) were lost to follow-up (19). Scarcity of specialists further exacerbates barriers to in-person hearing care (20). Quality audiology and otolaryngology specialty services exist in Alaska, but the vast majority are in Anchorage, while roughly half of the population resides in rural locations. Even in-person interactions with nurses, advanced practice providers, and primary care physicians are limited in rural communities, where recruitment of healthcare workers is a significant problem (21). As a result, rural children typically receive their healthcare from community health aide/practitioners at local village health clinics (21). Barriers associated with specialty follow-up care, exemplified by the challenges with follow-up after school hearing screening, have magnified health disparities in rural and marginalized populations (22–24).

**Figure 1:**
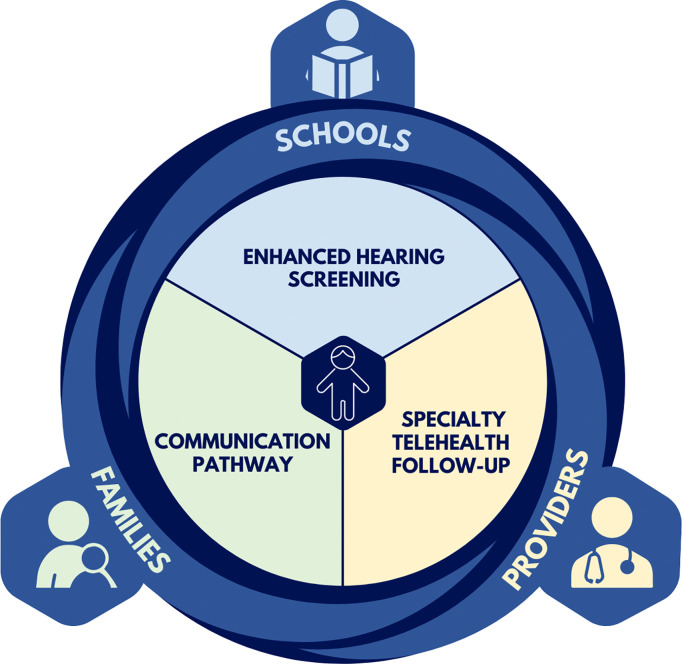
Core Components of the STAR Model

We will conduct a hybrid type 1 effectiveness-implementation randomized trial to evaluate the Specialty Telemedicine Access or Referrals (STAR) model, which is comprised of evidence-based enhanced hearing screening, school-based specialty telehealth follow-up, and streamlined communication between schools, healthcare providers, and families (Fig. 1). A preliminary version of this model was developed in a previous Patient-Centered Outcomes Research Institute (PCORI)-funded randomized trial that we conducted in rural Northwest Alaska (25, 26). That trial identified the most accurate screening process for rural Alaskan populations with a higher prevalence of middle ear disease and established the effectiveness of specialty telehealth follow-up for school hearing screenings compared to the usual referral process (25, 27, 28). An in-depth mixed methods evaluation highlighted that success of the telehealth follow-up intervention varied substantially between communities based on clinic capacity, personnel and ownership, awareness, and communication between schools and clinics (19, 29). A key recommendation that resulted from qualitative interviews was that specialty telehealth follow-up should be provided directly in the school setting instead of in local clinics (19, 21). Building on this work, the North STAR trial will adapt and implement the STAR model and evaluate effectiveness of a refined, school-based version of specialty telehealth follow-up in three distinct regions elsewhere in rural Alaska, with the goal of advancing the sustainability and scalability of the STAR model. Ultimately, we aim to create a novel, scalable model for school-based specialty telehealth follow-up that can be implemented statewide in Alaska and in other rural areas of the United States.

### Objectives {7}

**Figure 2:**
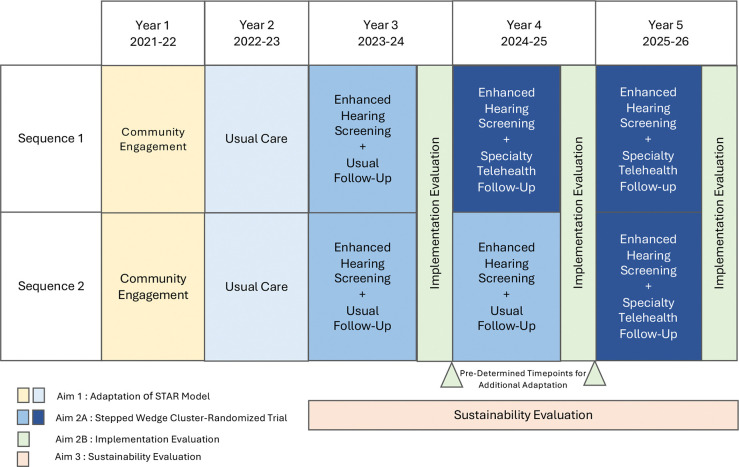
North STAR Trial Timeline

The North STAR trial will begin with a formative phase, in which we will collect data on the participating regions’ current school screening and follow-up practices, as well as stakeholder preferences and perspectives. We will use these data to systematically adapt the STAR model and to develop an implementation plan for use in the three regions participating in the trial, guided by the Consolidated Framework for Implementation Research (CFIR) **(Aim 1)** (Fig. 2) (30, 31). We will use this plan to implement the adapted STAR model in a stepped-wedge, cluster-randomized trial across three regions of Alaska. We will evaluate the effectiveness of specialty telehealth follow-up by measuring the proportion of referred children receiving specialty follow-up within 60 days of date of screening **(Aim 2a)**. Implementation of the model will be evaluated by measuring CFIR-based implementation determinants (innovation characteristics, inner setting, outer setting, individual characteristics, and implementation process) and implementation outcomes (fidelity, reach, acceptability, appropriateness, and feasibility) **(Aim 2b)** (31, 32). Sustainability of the STAR model will be evaluated via iterative meetings with state leaders, policymakers, and other relevant groups such as public and private payors, as well as through policy and procedure document review. From these meetings and reviews, we will identify key targets for policy and practice change to facilitate asynchronous school-based telehealth and develop materials to support decisionmakers regarding these policy and practice changes **(Aim 3)**.

### Trial design {8}

**Figure 3:**
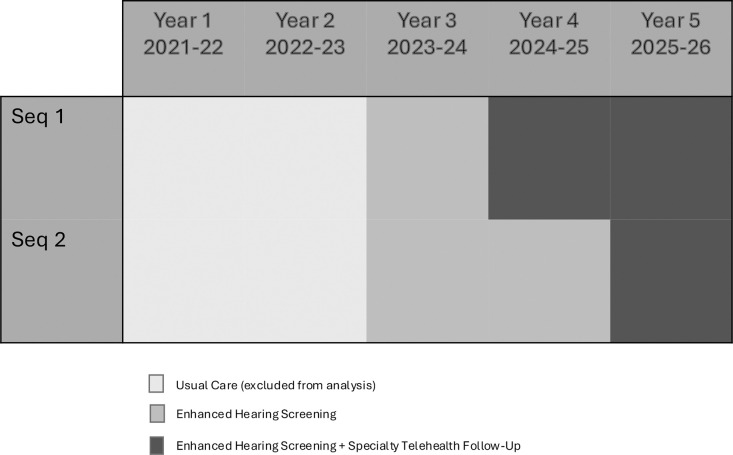
Stepped Wedge Trial Design

The North STAR trial is a two-sequence, 4 period, stepped wedge, cluster-randomized hybrid type I effectiveness-implementation trial which will be conducted in 3 regions of rural Alaska (Fig. 3). The trial will evaluate the STAR model, which consists of three core components: 1) enhanced school hearing screening, 2) school-based specialty telehealth follow-up, and 3) streamlined communication between schools, healthcare providers, and families.

## Methods: Participants, interventions and outcomes

### Study setting {9}

Spanning 586,000 square miles, the state of Alaska is equivalent to the entire US eastern seaboard, from Maine to Florida and west to Tennessee. Population density in this remote state is 70-times less than the national average, and much of the population lives in rural communities of less than 1000 people, while nearly 70% of physicians are located in the largest city of Anchorage (33, 34). Telehealth is essential in this environment where traditional patient-physician encounters require travel by plane (35, 36). In collaboration with leaders in the Alaska Department of Education and Early Development, three regions will be selected for the proposed initiative to be representative of the diversity across the state, including payor models, healthcare access, rurality, demographics, and geography. All three regions will participate in the formative phase.

### Eligibility criteria {10}

For adaptation of the STAR Model (Aim 1), participants will include educational staff, healthcare staff, parents, and children (n=30) who live in or are affiliated with participating communities. Eligibility will be determined regardless of race, gender, or ethnicity. Leaders in the Alaska Department of Education and Early Development will provide guidance to the study team on potential school districts that might participate in the stepped-wedge randomized trial (Aim 2a). The study team will engage with superintendents in these districts to gauge interest. In selected regions, all students enrolled in grades that the district typically screens for hearing will be eligible to participate regardless of age, gender, race, or ethnicity (n=~2000 children/year). For the implementation evaluation of the STAR model (Aim 2b), participants will include educational staff, healthcare providers, and parents involved in the implementation of the STAR model and will be eligible to participate regardless of age, race, gender, or ethnicity (n=~9–18). Lastly, for the sustainability evaluation of the STAR model (Aim 3), participants will include state leaders, policymakers, healthcare and education administrators, and Tribal leaders (n=~12–16). Eligibility will be determined regardless of age, race, gender, or ethnicity.

### Who will take informed consent? {26a}

Informed consent procedures and study enrollment for Aim 1 semi-structured interviews will be conducted by trained research staff either in-person (on paper) or remotely (electronically). Online consent forms will be stored and accessed via REDCap, a secure research survey platform. If informed consent is occurring remotely, a member of the study team will provide the potential participant a link to REDCap to access their consent form. Study staff will be available to answer any questions and will specifically ask the participant if they have any questions at the end of the consent process. A copy of the signed consent form will be offered to the participant.

Since hearing screenings are standard practice as part of annual school screening processes, parental permission and child assent for the hearing screening will be conducted per usual school district procedures during the trial (Aim 2a). For those children who receive a referral from hearing screening, parental consent will be required for participation in the specialty telehealth follow-up intervention. The timing and medium of the informed consent process will be developed based on feedback received from key informants in Aim 1 interviews. Written child assent will not be used, but should a child show unwillingness during the specialty telehealth follow-up (e.g., pushing the probe away, crying), the intervention will not be completed. For Aim 2b questionnaires and interviews, stakeholders will engage as partners instead of consented participants. All partners will be provided a participant information sheet which will outline what will be asked of them and emphasize the voluntary nature of their contribution. Study staff will document partners’ agreement to participate.

During the sustainability evaluation of the STAR model (Aim 3), state leaders, policymakers, and other relevant groups will be engaged as leaders and experts for this aim and will not engage as consented participants. Member checking will be used to ensure we have appropriately captured their feedback.

### Additional consent provisions for collection and use of participant data and biological specimens {26b}

N/A. No biological specimens will be collected.

### Interventions

#### Explanation for the choice of comparators {6b}

**Figure 4:**
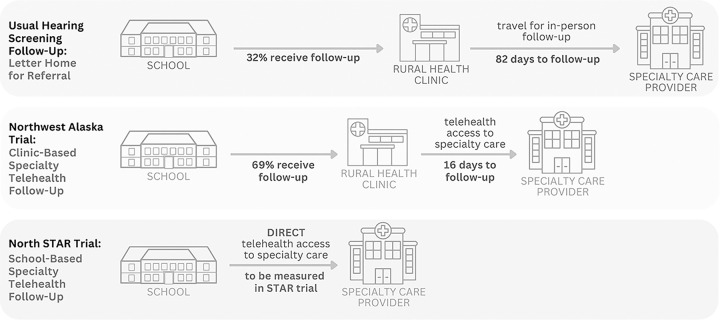
Comparison of Follow-Up Pathways Following School Hearing Screening Legend: Data for first two rows provided from our prior trial in northwest Alaska.

The trial intervention will be the STAR model, which consists of 1) enhanced school hearing screening, 2) school-based specialty telehealth follow-up, and 3) streamlined communication between schools, healthcare providers, and parents/caregivers. This intervention was designed based on evidence from our prior trial in Northwest Alaska, which provided critical proof of concept that telehealth can improve follow-up after school hearing screening (25). Because of the strong recommendation from the mixed methods component of the prior trial to bring telehealth technology into the school setting (19), this trial will evaluate the use of school-based specialty telehealth follow-up among children who receive a referral from school hearing screening (intervention) compared with the traditional referral, which is a letter home to parents advising follow-up (control) (Figure 4).

The enhanced hearing screening component, also developed based on our prior trial, is not randomized since it is based on established evidence. The enhanced hearing screening that will be implemented in all participating schools consists of a hearing assessment combined with tympanometry, a middle ear assessment that is essential for identifying preventable, infection-related hearing loss. Based on our prior data, children 7 years and older will receive pure-tone screening, while children 6 years and younger will receive otoacoustic emissions (OAE) screening. All children will receive tympanometry to assess middle ear health (27). The STAR enhanced hearing screening protocol differs from the screenings currently being done in Alaska. The state mandates school hearing screening, but protocols vary across school districts and do not include tympanometry (37).

- Pure-tone screening: will include screening at 1, 2, 4, and 6 kHz at 15 dB. Frequencies were selected based on evidence from our prior trial in Alaska (27), and the 15dB screening level was selected to align with the new World Health Organization definition of hearing loss (37). A tone will be presented at each frequency to each ear. If a child does not respond to a tone, a second tone will be played. Failure to respond to any tone twice at any frequency in either ear will generate a referral (39).
- OAE screening: will include screening at 2, 3, 4 and 5 kHz, with a 3 of 4 frequency pass criteria (40).
- Tympanometry screening: Type B (flat) tympanogram or negative pressure less than −200 decapascal (daPa) will generate a referral per standard clinical guidance (41).

The communication component between schools, healthcare providers, and parents/caregivers will be developed during the systematic adaptation of the STAR model in Aim 1 and rolled out with specialty telehealth follow-up. .

#### Intervention description {11a}

The trial will evaluate the effectiveness of school-based specialty telehealth follow-up. Enhanced hearing screening and specialty telehealth follow-up will be designed to use a single unified platform. It will include tools for initial screenings (OAE, tympanometry, and pure-tone screening), as well as a digital otoscope. Results will be transmitted asynchronously to an audiologist, who will determine the recommended plan of care. This process will be conducted onsite at the school. Parents will be notified that the specialty telehealth follow-up is occurring but will not be required to attend, based on community feedback in our prior trial (21, 29).

#### Criteria for discontinuing or modifying allocated interventions {11b}

This study entails minimal risk, and adverse events are not expected. Participant involvement includes a one-time enhanced hearing screening that will take about 5–10 minutes, and for children who receive a referral from screening, a one-time, 15–20-minute session to collect ear and hearing information for specialty telehealth follow-up. We do not expect discontinuation from the specialty telehealth follow-up intervention; however, if a parent/caregiver chooses to withdraw their child from the study, we will stop all study related activities not yet completed.

#### Strategies to improve adherence to interventions {11c}

We will develop training materials and an implementation plan for the STAR model during Aim 1. Throughout the trial, we will track fidelity to the implementation plan, as well as adaptations. These measurements will be taken annually, as part of the implementation evaluation activities. To reduce burden on school staff conducting the screenings, fidelity will primarily be assessed by the research team using a structured fidelity checklist, which will be developed to align with the implementation plan. Checklist data will be analyzed as part of the mixed methods evaluation for Aim 2b.

#### Relevant concomitant care permitted or prohibited during the trial {11d}

As a pragmatic trial, we will not prohibit concomitant care. Parents and their children will be permitted to seek their own hearing care and arrange for follow-up or other interventions as they choose.

#### Provisions for post-trial care {30}

N/A. This trial specifically addresses follow-up immediately after school hearing screening and not subsequent care.

#### Outcomes {12}

The primary effectiveness outcome will be the proportion of referred children (i.e., those who failed screening) who receive specialty follow-up within 60 days of screening date. Secondary implementation outcomes include fidelity, reach, acceptability, appropriateness, and feasibility. An additional secondary outcome will be sustainability. Outcome details are included in Table 1.

#### Participant timeline {13}

The timeline for the trial is detailed in Figure 2 and Table 2. Broadly, the formative phase to adapt the STAR model (Aim 1) will be conducted during Years 1–2. The enhanced hearing screening portion of the STAR model will be implemented in all participating schools in Year 3, and Year 3 will serve as baseline data for the stepped wedge, cluster randomized trial (Aim 2a). Specialty telehealth follow-up will be implemented in a phased fashion during Years 4 and 5 based on sequence randomization. The implementation evaluation (Aim 2b) will be conducted annually in Years 3–5, and the sustainability evaluation (Aim 3) will occur over the same timeframe.

#### Sample size {14}

The stepped-wedge cluster randomized trial will be conducted in approximately 25 schools located in 3 regions of rural Alaska. For power calculations, we used enrollment numbers from school districts in 3 regions representative of the regions that will be considered for inclusion in the trial. These 3 regions serve a combined total of approximately 2,072 students per year in grades that perform hearing screening, with a median of 99 students per school per year.

The study design is open cohort; however, in practice, we expect it to more closely resemble a repeated cross-sectional design because we expect most data to be collected from different children in each year of the study due to existing school protocols of not screening every grade. Therefore, for the purposes of calculation of power we conservatively assumed a repeated cross-sectional stepped wedge design with a binary outcome. To do so, we used the approach from Hemming et al. based on a comparison of two proportions (44), incorporating design effects to account for clustering by school, repeated measures on the same cluster and t-based inference to accommodate the small number of clusters (i.e. 25 schools).

**Figure 5:**
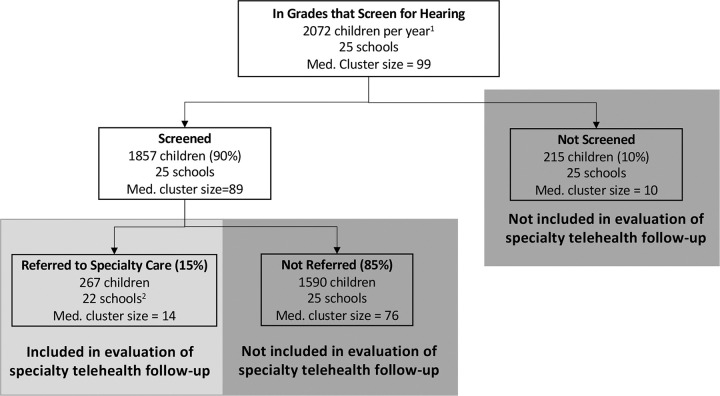
Estimating Cluster Size for Primary Effectiveness Outcome Based on Prior School Data ^1^ Based on 2020–2021 enrollment data, assume similar enrollment level across all cluster periods and the following screening practices: Region 1 screens K-5; Regions 2 and 3 screen K, 1, 3, 5, 7, 10. ^2^ Based on school enrollment, assumed screening rate of 90%, and assumed referral rate of 15%, 3 schools are expected to have zero referred children.

To calculate the expected cluster size for the primary outcome, it was necessary to estimate the number of referred students for each year in each school. To do this, we multiplied an assumed school-level screening rate of 90% (based on experience from our prior trial) by the 2020–2021 school-level enrollment in grades that perform hearing screening (based on local knowledge of previous screening protocols in each region) and by an expected referral rate of 15%. The expected 15% referral rate was based on historical, regionwide referral rates from the 2020–2021 and 2021–2022 school years. Estimated cluster sizes were then rounded down to the nearest whole number to obtain the final expected cluster size for the primary outcome (Figure 5). Enrollment counts in eligible grades in each region were also used to estimate the coefficient of variation (CV) of cluster size to generate appropriately conservative power estimates accounting for variation in cluster size.

Using an expected referral rate of 15%, estimation of median cluster size revealed that 3 of the smaller schools may have zero referrals. Among the remaining 22 of 25 schools that are expected to have referrals, we anticipate a median cluster size of 14 referred children and a CV of cluster size of 0.59. For simplicity, we also assumed an equal number of schools per sequence (11 schools). Regarding the rationale for our assumed proportion of referrals resulting in specialty telehealth follow-up within two months, we note that in our prior trial we observed a 42.2% percentage point difference in number of children seen for specialty follow-up within 60 days of screening (25, 28). To be conservative, we assumed the proportion with specialty follow-up to be 20% for children in the control condition and an expected 40 percentage point difference in follow-up between treatment and control conditions. We estimated a within-period intracluster correlation (ICC) of 0.30, based on the results of our prior trial and conservatively assumed a cluster autocorrelation (CAC) of 0.7 with a two-period decay (i.e., nested exchangeable) structure between measurements within the same cluster over subsequent time periods. We assume a two-period decay structure in the design phase rather than an alternate less realistic structure such as exchangeability to protect against an underpowered trial (45,46). With these assumptions, and an alpha of 0.05, the trial will have greater than 90% power to detect an increase of at least 40 percentage points in the proportion of referred children receiving specialty follow-up (comparable to the 42-percentage point difference seen during the prior trial) (46–48).

**Figure 6:**
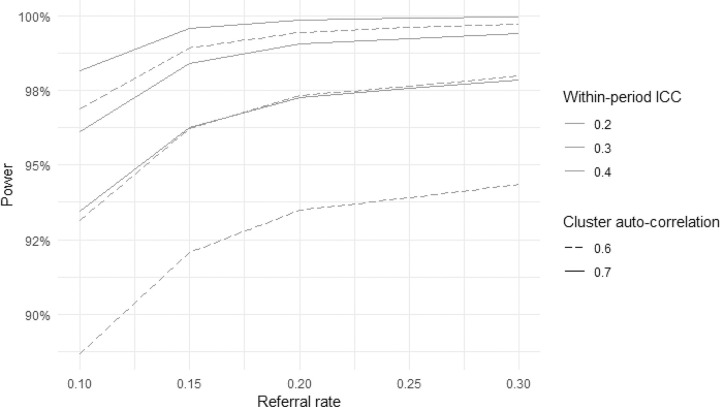
Sensitivity Analysis for Primary Effectiveness Outcome Assumptions: Screening rate: 90%; Control follow-up rate: 20%; Intervention follow-up rate: 60%. Screening K-5 in Region 1; Screening K, 1, 3, 5, 7, 10 in Regions 2 and 3. Number of clusters, median cluster size, and CV of cluster size dependent on referral rate.

A sensitivity analysis was performed to assess the effect of changes in assumptions for referral rate, within-period ICC, and cluster autocorrelation (CAC) on power for the primary outcome analysis (Figure 6). Using 80% power as minimum target power (and 90% as preferred power level), the plots show acceptable power in all scenarios.

#### Recruitment {15}

Participants in Aim 1 will include educational staff, healthcare providers, parents, and children in the three regions participating in the study. The number of participants to be recruited will be based on the dual goal of reaching saturation and achieving representation of several different types of educational staff and healthcare providers, different regions, and different school sizes. Potential participants will be identified using purposeful and/or snowball sampling. Recruitment outreach will occur via email, flyers, presentations, and/or in-person, as appropriate. Informed consent procedures and study enrollment will be conducted by trained research staff. Children must have a parent/caregiver provide informed consent for a child to participate. In addition, children aged 7 to 17 years will be required to sign a child assent form. It is unlikely that a child under age 7 will participate. However, should a child younger than 7 years wish to enroll, then a verbal assent will be used in combination with parent/guardian informed consent.

Leaders in the Alaska Department of Education and Early Development will provide guidance to the study team on potential school districts that might participate in the randomized trial (Aim 2a). The study team will engage with superintendents in these districts to gauge interest. In participating regions, all students enrolled in grades that the district typically screens for hearing will be eligible for hearing screenings. Per standard practice, parents may opt out of school hearing screenings if they prefer their child not to receive screening. All students who receive a school hearing screening referral will be eligible for specialty telehealth follow-up (Years 4–5), but parent/caregiver consent will be required. Consent forms for the specialty telehealth follow-up will be included in routine annual school paperwork, or in other mediums based on guidance by the Community Advisory Board (CAB) and community input obtained during Aim 1.

Participants in the STAR model, including school staff, healthcare providers, and parents, will be engaged in questionnaires and interviews to evaluate implementation (Aim 2b). These individuals will participate as partners instead of consented research participants. Study staff will use email, phone calls, or in-person interactions to invite these partners to contribute. A participant information sheet will be provided to partners which emphasizes the voluntary nature of their contribution.

Relevant policymakers, state leaders, decision-makers in state government in healthcare, healthcare financing, and education; leaders of Tribal community and healthcare entities; and leaders of regional or local organizations that work on rural healthcare delivery will be recruited to participate in the sustainability evaluation of STAR (Aim 3). The sampling frame and specific leaders contacted to participate will be established through guidance from regional leadership and the CAB. Leaders will be recruited to participate as partners instead of consented research participants. A participant information sheet will be provided to partners which emphasizes the voluntary nature of their contribution.

We do not anticipate challenges with recruitment for the trial because data collection will occur at the population level, with entire schools participating. Individual consent will be obtained for the specialty telehealth follow-up intervention, and a community-informed implementation plan will be developed during the formative phase in Aim 1 to maximize uptake.

### Assignment of interventions: allocation

#### Sequence generation {16a}

Schools (n~25) will be randomized to one of two sequences using constrained randomization (49). Constrained randomization will be used to balance important baseline covariates between the two sequences in order to increase statistical power and improve internal validity of the trial. This will involve calculating a balance score for each allocation, incorporating into the balance metric candidate covariates expected to be correlated with the primary outcomes (chosen a priori by study team consensus), including: region, grades served by the school, hub school vs rural school, school enrollment (from 2022–2023 school year), proportion of students qualifying for free or reduced lunch, and baseline screening rate.

#### Concealment mechanism {16b}

Independent statisticians will perform the final randomization, and study statisticians will remain masked to allocation until the primary analysis is complete. The allocation cannot be masked for other study team members, school staff or parents for logistical reasons, but allocation will only be revealed when required.

#### Implementation {16c}

The independent statistician who performs the randomization will share the allocation with members of the study team responsible for implementing the intervention. Entire schools will participate in the trial; therefore, individual students are not enrolled or assigned to interventions.

### Assignment of interventions: Blinding

#### Who will be blinded {17a}

Statisticians will be blinded to allocation until primary analysis is complete. Allocation will only be revealed to study team members who require this information to manage the logistics of implementation at participating schools. School staff and parents will not be blinded to allocation.

#### Procedure for unblinding if needed {17b}

N/A. Families are not blinded..

### Data collection and management

#### Plans for assessment and collection of outcomes {18a}

The STAR model is comprised of enhanced hearing screening, specialty telehealth follow-up, and streamlined communication between schools, healthcare providers, and parents/caregivers. As described earlier, the enhanced hearing screening component of the intervention is based on prior evidence from our previous trial in Northwest Alaska and will not be randomized (27). As such, enhanced hearing screening will be implemented in all participating schools in Year 3 of the study, and Year 3 will be considered baseline for measurement of the primary effectiveness outcome. Specialty telehealth follow-up will be implemented in a stepwise fashion in Years 4 and 5 of the trial, with half of participating schools receiving specialty telehealth follow-up in Year 4 and all schools receiving it in Year 5.

The primary effectiveness outcome for the trial (Aim 2a) will be measured using annual queries of electronic health records (EHR) from the healthcare systems serving each region conducted (Table 1). We will engage with regional general medical centers, audiology practices, ENT practices, and Native health organizations in selected regions to gain a comprehensive understanding of clinical options available for follow-up care, and these regional sources will be included in the EHR queries. School screening data will be linked with queries from regional EHR and deidentified via a HASH ID process. Students with no record of receiving specialty care within 60 days of their date of screening will be considered not to have received follow-up care, as we will not be aware of students who move out of the region or receive follow-up care elsewhere. Based on our previous trial in Northwest Alaska, collecting EHR data for 60 days from screening date is sufficient to determine differences in rates of follow-up (25).

Secondary implementation outcomes (Aim 2b) will include fidelity, reach, acceptability, feasibility, and appropriateness (Table 1) (32). Fidelity will assess the extent to which core components of STAR are implemented as intended. Reach will assess the proportion of students reached by the STAR model. Acceptability, feasibility, and appropriateness will evaluate satisfaction with the STAR model and perceived fit of the intervention among participating school staff. Quantitative data sources for implementation outcomes will include trial process records, validated questionnaires, and school/regional-level data. Qualitative data sources will include interviews or focus groups with a subset of educational staff, healthcare providers, and parents participating in STAR. Qualitative interviews will occur annually during Year 3 (2023–24), Year 4 (2024–25), and Year 5 (2025–26) at the conclusion of school hearing screening (Figure 3). Specific measures used for each implementation outcome are detailed below.

Fidelity will be measured with structured checklists assessing adherence to the core components of the intervention. Individual components of the checklist will be designed during the formative phase. Fidelity of the telehealth specialty follow-up portion of the STAR model will also be evaluated by assessing time to specialty follow-up, comparing the date of specialty ear/hearing encounter to the date of screening. This measure will differ from the primary effectiveness outcome in that it will evaluate time to follow-up as a numeric variable rather than a categorical variable (i.e., follow-up within 60 days or not). Semi-structured interviews with educational staff, healthcare providers, and parents will be used to further explore contextual factors that influence variation in fidelity between schools. For educational staff, probes will be developed to ask about how various facets of the STAR intervention were used (i.e., which grade levels were screened, what screening tests were used, referral criteria, parental communication method). Similarly, for healthcare staff, probes may ask about whether follow-up care was completed using the telehealth interface developed for the STAR intervention.

Reach will be assessed annually via deidentified screening data from each region. This will include proportion of children screened using the STAR model by grade and school, focusing specifically on grades that typically receive screening in each individual school. We will also evaluate representativeness of children reached by the STAR model based on demographic factors such as race/ethnicity and region.

For acceptability, feasibility, and appropriateness, key informants will complete validated questionnaires, including Intervention Appropriateness Measure (IAM), Acceptability of Intervention Measure (AIM), Feasibility of Intervention Measure (FIM) (42), and Organizational Readiness for Implementing Change (ORIC) (43). Educational staff informants serving multiple schools will be asked to complete the survey for the region they are serving. Surveys will be completed annually to differentiate outcomes across various STAR components as they are rolled out. Annual qualitative semi-structured interviews with educational staff, healthcare providers, and parents will also be used to assess the acceptability, feasibility, and appropriateness of the intervention.

Sustainability of the STAR model (Aim 3) will be assessed qualitatively through iterative discussions with state leaders, policymakers, payors, and other relevant groups as well as through policy and procedural document review. From these discussions, we will identify key targets for policy and practice change and develop materials to support decisionmakers regarding these policy and practice changes.

#### Plans to promote participant retention and complete follow-up {18b}

Loss to follow-up is not expected to be not applicable for this study design, which involves a one-time, state-mandated school hearing screening and a one-time specialty telehealth follow-up for referred children. Follow-up data will be obtained from regional EHR queries with deidentification using HASH ID, thus reducing risk for loss to follow-up.

#### Data management {19}

The University of Arkansas for Medical Sciences (UAMS) will be the data coordinating center and will oversee data architecture and management. A secure research database will house all Aim 2a trial data from schools and healthcare entities. Data from other aims will be stored in password-protected, HIPAA-compliant cloud server hosted by Southcentral Foundation (SCF) and/or UAMS.

Schools in the participating regions will receive STAR model screening kits with telehealth capability. Schools will maintain their usual practice for tracking and managing school hearing screening results. All school and healthcare participant data will be deidentified via a HASH ID process prior to being shared with the research team. This HASH ID process will irreversibly remove identifiers (e.g., name, address) from the data while still allowing linkage of limited data sets from the school and healthcare systems. After this process, data will only be labelled by unique identification numbers. All data will be reported in aggregate, and no individual identifiers will be used in reporting.

Nearly all source documents will be electronic in nature and stored in cloud-based servers. All effort will be made to limit locally stored data, with copies backed up to secure servers and/or cloud-based solutions. Standard quality assurance measures will be carried out by the data manager and will be overseen by the Principal Investigators to ensure data quality and detection of erroneous data in accordance with the Data Safety Monitoring Plan (DSMP). Data quality control will be performed largely by the biostatistical team.

#### Confidentiality {27}

Participant confidentiality and privacy will be strictly held in trust by the study team. This confidentiality will be extended to the data being collected as part of this study. Data that could be used to identify a specific study participant will be held in strict confidence within the research team. No personally identifiable information from the study will be released to any unauthorized third party without prior written approval from the ethical oversight boards that will be overseeing the study.

Data will be collected and stored in password-protected HIPAA-compliant cloud-based systems (REDCap, OneDrive, and Box), with access restricted to study team members who need access to the data to fulfill their job duties. Most data will be captured electronically. Any data that are collected initially on paper or non-digital format will be locked in a file cabinet within a key-accessed office at SCF or UAMS. Confidentiality of participant data for the trial will be ensured by de-identification of child-level data using the HASH ID deidentification process. The PIs will ensure all mechanisms used to share data will include proper plans and safeguards for the protection of privacy, confidentiality, and security for data dissemination and reuse (e.g., all data will be thoroughly de-identified and will not be traceable to a specific study participant). The adequacy of the Research Data Security Plan will be evaluated and approved by SCF and UAMS. Any publications or presentations that result from this research will not identify any subjects individually and will present data in aggregate form only.

#### Plans for collection, laboratory evaluation and storage of biological specimens for genetic or molecular analysis in this trial/future use {33}

Not Applicable. There will be no collection of biological specimens in this trial.

### Statistical methods

#### Statistical methods for primary and secondary outcomes {20a}

For analysis of the primary outcome (proportion of referred children who receive specialty follow-up within 60 days of screening date), the intention-to-treat (ITT) population will be all children referred for follow-up during their school’s hearing screening period, over Years 3–5, regardless of whether their parent/guardian opts in to the specialty telehealth follow-up intervention.

Child-level outcomes will be analyzed using generalized estimating equations (GEE) in order to accommodate correlation within schools that is expected to arise due to the repeated cross-sectional data obtained over time. Moreover, the GEE approach will estimate population-averaged intervention effects that are relevant to public health implementation (50, 51). More specifically, binary outcomes at the child level will be analyzed assuming a binomial outcome distribution and will include the following terms: an indicator for whether the school (i.e. cluster) is observed under the usual care or intervention condition during the cluster-period; time effects to account for the potential confounding effect of time, fixed effects for the variables used in the constrained randomization procedure; and pre-specified adjustment for sociodemographic characteristics (e.g. age, gender, race, ethnicity, and free-reduced lunch eligible) of children to account for potential confounding.

Results will be reported on the relative scale as risk ratios, and absolute effects will be reported as risk differences, both with 95% confidence intervals (52). To do this, the identity and log-link will be used to estimate absolute and relative effects, respectively. In the event of issues with convergence of either the log-binomial or identity-binomial GEE models, we will use the modified-Poisson approach with log or identity link, as needed. If further convergence issues arise with the log-link, we will instead use the standard logit-binomial GEE and report odds ratios as the estimate a relative effect, and if further convergence issues arise with the identity link, the Gaussian distribution will be used with identity link to estimate absolute effects. Standard errors will be estimated using the robust sandwich variance estimator using appropriate small-sample corrections to obtain valid standard errors (53). The GEE estimation approach assumes that data are missing completely at random (MCAR), thus this assumption will be checked. ICC will be calculated to quantify the extent to which outcomes vary by school.

#### Interim analyses {21b}

No interim analyses are planned for the primary trial outcome. As a result, there are no stopping guidelines.

#### Methods for additional analyses (e.g. subgroup analyses) {20b}

The choice of variables around which to perform sub-group analyses will be informed by results from preliminary qualitative analyses as well as clinical knowledge around factors that are reasonably expected to influence treatment effect heterogeneity (HTE). From a list of potential candidate variables, sub-group analyses will move forward contingent on the presence of substantial variation in that variable present during baseline data collection. Sub-group analyses will be prespecified as part of the detailed analysis SAP. Each subgroup variable will be included as a fixed effect and interacted with the treatment indicator variable. Contrast statements will be used to produce stratified estimates by subgroup. Where possible, covariate ICCs will be calculated for subgroup variables analyzed for HTE.

#### Methods in analysis to handle protocol non-adherence and any statistical methods to handle missing data {20c}

Missing data may occur if referred children move out of the participating regions or receive follow-up care from providers outside our data observation scope. Child-level enrollment date ranges will help us determine if the former has happened, but we cannot track the latter. Therefore, our analysis will assume that if a specialty follow-up visit is not recorded in our data (and the child remains enrolled in a regional school), the visit did not take place. Depending on the pattern of missingness at the school level, we may employ an inverse probability weighting or multiple imputation approach. We will use complete case analysis only if data loss is thought to be missing completely at random (MCAR).

#### Plans to give access to the full protocol, participant level-data and statistical code {31c}

All finalized datasets, protocols, and study materials will be made publicly available upon request with necessary approvals and execution of a formal data use agreement. The PIs will ensure all mechanisms used to share data will include proper plans and safeguards for the protection of privacy, confidentiality, and security for data dissemination and reuse.

### Oversight and monitoring

#### Composition of the coordinating centre and trial steering committee {5d}

N/A. Trial Steering Committee is not applicable for this minimal risk, pragmatic trial.

#### Composition of the data monitoring committee, its role and reporting structure {21a}

N/A. Data Monitoring Committee is not applicable for this minimal risk, pragmatic trial. A DSMP is in place.

#### Adverse event reporting and harms {22}

The PIs and study staff will develop procedures for data collection, data entry, and quality checks. Any data anomalies will be communicated to the site(s) for clarification. Following written standard operating procedures (SOPs), the PIs will verify that data are generated, documented, and reported in compliance with the protocol and the applicable regulatory requirements for Good Clinical Practices (GCP).

No adverse events are anticipated from the intervention. The equipment will be designed for lay-person use, and adequate training and support will be provided. The enhanced hearing screening and specialty telehealth follow-up will introduce no more than ordinary risk that is encountered in routine school hearing screening and follow-up. For qualitative procedures and data collection, no adverse events are anticipated. Should an individual experience any sensitivities during qualitative data collection, study staff will be trained for an appropriate response, including concluding the data collection event early. An iterative process will be put into place to adapt interview questions and study personnel training as needed to address this possible risk.

Any adverse events or unanticipated problems will be documented and reported within 24 hours to the PIs. If required, PIs will report the adverse event or unanticipated problem to the Institutional Review Board (IRB) of record within 7 days and will determine necessary action, from changes to the protocol to cessation of activity, in compliance with IRB oversight.

#### Frequency and plans for auditing trial conduct {23}

Interval quality assurance audits will be conducted to ensure data quality and detection of any erroneous data. Data exports, integrated equipment, and secure cloud-based solutions will be used to ensure collection of high-quality data at each participating school. Site monitoring will also ensure that the rights of human participants are protected, that the reported trial data are accurate, complete, and verifiable, and that the trial is being conducted in compliance with the approved protocol/amendment(s). Monitoring will be conducted using web-based data validation rules and data manager review.

#### Plans for communicating important protocol amendments to relevant parties (e.g. trial participants, ethical committees) {25}

Important protocol modifications will be communicated to the IRB of record, participating communities, trial registration, and the funder. The CAB will guide communication with communities, including relevant school and healthcare entities.

#### Dissemination plans {31a}

This study will comply with the NIH Data Sharing Policy and Policy on the Dissemination of NIH-Funded Clinical Trial Information and the Clinical Trials Registration and Results Information Submission rule. The trial is registered at ClinicalTrials.gov, and results from this trial will be submitted to ClinicalTrials.gov. All abstracts and manuscripts produced from this research will be submitted for review at Southcentral Foundation and other local entities as appropriate. Community dissemination efforts will be prioritized at the end of the trial to communicate study findings to members of each of the involved communities. We will also make our results available to the global scientific community and welcome the input and collaboration of others studying similar scientific questions in this rapidly moving discipline. Overall, the sharing of data generated by this project will be carried out through academic presentations at regional, national, and international scientific meetings, publication in peer-reviewed journals, and local dissemination in the form of presentations at local community townhalls, school programs, and other venues in according with CAB guidance. Layperson summaries of findings will be published in local newsletters and social media. Local dissemination will be directed by the CAB and regional stakeholders. Priority will be placed on making all publications from this work open access to facilitate community accessibility. Final accepted manuscripts will be deposited into PubMed Central in compliance with the NIH Public Access Policy.

## Discussion

The North STAR trial will evaluate a school-based telehealth intervention that has the potential to substantially improve access to specialty hearing care for rural children. Most childhood hearing loss in rural areas is preventable, but access to the necessary specialty care from audiologists and otolaryngologists in rural areas is limited. While school health programs such as hearing screening provide critical access to preventive care for rural children, loss to follow-up is a pervasive problem. Without intervention, hearing loss has broad-reaching implications for educational attainment, vocational opportunities, and quality of life (1, 3–10). Thus, innovations that can connect rural children to specialty follow-up have the potential to address serious lifelong consequences of untreated childhood hearing loss.

This trial is built on key evidence already developed in the state of Alaska, which determined the optimal methods of screening children for hearing loss in environments where ear infections are common, as well as the vital role that telehealth can play in improving follow-up after screening (13, 25, 27, 28). Both the North STAR trial and our preceding trial in northwest Alaska involve inclusive designs that prioritize community engagement and feedback (21, 29). The STAR model was developed directly from community feedback from the prior trial indicating that transition of specialty telehealth follow-up from clinic to a school-based setting will be essential for improving consistent implementation and long-term success of the intervention (19. 29). Based on CAB guidance, the enhanced screening component of the STAR model is not randomized in this trial, as clear evidence was already developed in another region of Alaska indicating the most accurate screening protocols by age (27). This trial will therefore implement the new evidence-based screening protocol in all participating schools prior to rollout of specialty telehealth follow-up. The STAR model will be refined based on community input during the formative phase of this trial, and ongoing feedback will be solicited during the implementation evaluations conducted annually throughout the trial, with predetermined timepoints for adaptation built into the study timeline to accommodate any needed community-informed modifications.

There are limitations to this trial that should be mentioned. This is a pragmatic trial conducted in a real-world setting, with school staff implementing the intervention. While this can pose challenges with fidelity and uptake, it is more reflective of reality for an intervention that is intended to be scaled across school districts and entire states. Our implementation outcomes will be essential for discerning community-level factors influencing success of the intervention. Cluster randomization inherently reduces power compared to a parallel design, but this design choice was necessary because the intervention is intended to be implemented in a school-wide fashion. Another limitation inherent in the study design is that it is not possible to blind participating schools and parents to allocation. A tremendous amount of coordination will be required with participating schools to ensure that they receive STAR equipment, the needed trainings occur, and materials informing parents of the program are provided for school enrollment packets. To mitigate this limitation, the statistical team will be blinded until primary analysis is complete, and allocation will only be revealed to study staff who need the information for logistics of deploying the intervention.

A key focus of the North STAR trial is sustainability. Emphasis will be placed on developing materials to support policy or practice changes identified by state leaders as valuable for facilitating sustained implementation of STAR. The goal of this trial is to develop a feasible, evidence-based model for school-based specialty telehealth follow-up that can be scaled across the state of Alaska and to other rural environments. Importantly, the STAR model has the potential to support not only increased access to specialty hearing care, but also expanded access to care for other preventable health conditions as well, providing a valuable tool for improving health equity for all rural children.

### Trial status

The grant supporting this trial was funded in September 2021, and baseline data collection for the trial began in September 2023. Trial completion is anticipated to be June 2026. Protocol version number 1.0, 8/28/2023.

## Figures and Tables

**Table 1.** 

Outcome	Specific Measurement Variable	Analysis Metric (Level of Analysis)	Aggregation Method	Measurement Timepoint for Analysis^#^
**Primary Outcome**				
**Primary Effectiveness Outcome**	Specialty follow-up received after referred screening	Final value (child)	Proportion	Within 60 days of screening date; Control condition will be collected in Year 3 for Sequence 1 and 2; Specialty telehealth follow-up condition will be collected in Years 4–5 for Sequence 1 and Year 5 for Sequence 2
**Secondary Outcomes**				
**Fidelity**	1. Adherence checklists 2. Time to specialty follow-up 3. Qualitative interviews	1. Final value (school) 2. Time to event (child) 3. Content analysis of qualitative data	1. Proportion of schools using STAR enhanced screening* 2. Median days to specialty follow-up from screening date 3. Contextual factors influencing fidelity between schools	Annually during Years 3–5
**Reach**	Children screened using the STAR model	Final value (school)	Proportion, overall and by race, ethnicity, region	Annually during Years 3–5
**Acceptability**	1. Acceptability of Intervention Measure (AIM) 2. Qualitative interviews	1. Final value (region) 2. Content analysis of qualitative data	1. Mean score 2. Themes	Annually during Years 3–5
**Appropriateness**	1. Intervention Appropriateness Measure (IAM)			
	2. Qualitative interviews			
**Feasibility**	1. Feasibility of intervention measure (FIM) and Organizational Readiness for Implementing Change (ORIC) 2. Qualitative interviews			
**Sustainability**	Sustainability of policies, payment, and infrastructure	1. Content analysis of qualitative data 2. Policy and procedure document review	1. Proposed policy targets for staff capacity, funding, and infrastructure 2. Materials to support decisionmakers	Years 3–5

# Year 3 is the 2023–2024 academic year; Year 4 is the 2024–2025 academic year; Year 5 is the 2025–2026 academic year.

* Adherence checklists will be designed during the formative phase. An example outcome is provided to be illustrative of types of data that will be collected from these checklists.

**Table 2.** 

	STUDY PERIOD			
	Enrollment	Pre-Allocation	Allocation	Post-Allocation
TIMEPOINT	*−t_2_* Year 1	*−t_1_* Year 2	*t_0_* Year 3	*t_1_* Year 4	*t_2_* Year 5
**ENROLLMENT**					
School District Eligibility Screen	X				
School District Invitation to Participate	X				
Individual Treatment Consent^$^				X^1^	X^1,2^
**ALLOCATION**			X		
**INTERVENTIONS**					
Adaptation of STAR Model	X	X			
Enhanced Hearing Screening			X	X	X
Usual Follow-up			X^1,2^	X^2^	
Specialty Telehealth Follow-up				X^1^	X^1,2^
**ASSESSMENTS**					
Child Demographics*			X	X	X
Hearing Screening Results*			X	X	X
Specialty Follow-up*			X	X	X
Implementation Outcomes^			X	X	X

Superscript used to denote whether activity applies to schools allocated to Sequence 1 (X^1^) or Sequence 2 (X^2^).

$ Since annual hearing screenings are standard practice, parental permission and child assent will be conducted per usual school district procedures during the trial. Treatment consent is required for the specialty telehealth follow-up intervention.

* De-identified data collected at the state level.

^ Implementation outcomes include: fidelity, reach, acceptability, appropriateness, feasibility, and sustainability. See Table 1 for specific variables and metrics for secondary implementation outcomes.

## Data Availability

The statistical team and PIs will have access to the final trial dataset. Access to the final dataset will be provided to other study team members if required to complete their job duties.

## Abbreviations

CAB: Community Advisory Board
CAC: Cluster Autocorrelation
CFIR: Consolidated Framework of Implementation Research
CONSORT: Consolidated Standards of Reporting Trials
CV: Coefficient of Variation
DSMP: Data Safety Monitoring Plan
EHR: Electronic Health Record
ENT: Ear Nose Throat physician (Otolaryngologist)
GCP: Good Clinical Practice
GEE: Generalized Estimating Equations
HIPAA: Health Insurance Portability and Accountability Act
HTE: Heterogeneity of Treatment Effects
ICC: Intracluster Correlation Coefficient
ICH: International Council on Harmonisation
IRB: Institutional Review Board
IT: Information Technology
IQR: Interquartile Range
ITT: Intention to Treat
MAEE: Matrix-Adjusted Estimating Equations
MDE: Minimum Detectable Effect
M-PI: Multiple Principal Investigators
NIH: National Institutes of Health
OAE: Otoacoustic Emission
OR: Odds Ratio
PCORI: Patient-Centered Outcomes Research
PI: Principal Investigator
REDCap: Research Electronic Data Capture
RR: Risk Ratio
SAE: Serious Adverse Event
SCF: Southcentral Foundation, Alaska
SD: Standard Deviation
SOP: Standard Operating Procedure
STAR: Specialty Telemedicine Access for Referrals
UAMS: University of Arkansas Medical Sciences
UP: Unanticipated Problems
US: United States
WHO: World Health Organization
